# An eigenvalue localization set for tensors and its applications

**DOI:** 10.1186/s13660-017-1331-1

**Published:** 2017-03-09

**Authors:** Jianxing Zhao, Caili Sang

**Affiliations:** College of Data Science and Information Engineering, Guizhou Minzu University, Guiyang, Guizhou 550025 P.R. China

**Keywords:** 15A18, 15A69, 65F10, 65F15, $\mathcal{M}$-tensors, nonnegative tensors, minimum eigenvalue, localization set

## Abstract

A new eigenvalue localization set for tensors is given and proved to be tighter than those presented by Li *et al*. (Linear Algebra Appl. 481:36-53, 2015) and Huang *et al*. (J. Inequal. Appl. 2016:254, 2016). As an application of this set, new bounds for the minimum eigenvalue of $\mathcal{M}$-tensors are established and proved to be sharper than some known results. Compared with the results obtained by Huang *et al*., the advantage of our results is that, without considering the selection of nonempty proper subsets *S* of $N=\{1,2,\ldots,n\}$, we can obtain a tighter eigenvalue localization set for tensors and sharper bounds for the minimum eigenvalue of $\mathcal{M}$-tensors. Finally, numerical examples are given to verify the theoretical results.

## Introduction

For a positive integer *n*, $n\geq2$, *N* denotes the set $\{1,2,\ldots ,n\}$. $\mathbb{C}$ (respectively, $\mathbb{R}$) denotes the set of all complex (respectively, real) numbers. We call $\mathcal{A}=(a_{i_{1}\cdots i_{m}})$ a complex (real) tensor of order *m* dimension *n*, denoted by $\mathbb{C}^{[m,n]}(\mathbb {R}^{[m,n]})$, if

$$a_{i_{1}\cdots i_{m}}\in{\mathbb{C}}(\mathbb{R}), $$

where $i_{j}\in{N}$ for $j=1,2,\ldots,m$. $\mathcal{A}$ is called reducible if there exists a nonempty proper index subset $\mathbb{J}\subset N$ such that

$$a_{i_{1}i_{2}\cdots i_{m}}=0, \quad \forall i_{1}\in\mathbb{J}, \forall i_{2},\ldots,i_{m}\notin\mathbb{J}. $$

If $\mathcal{A}$ is not reducible, then we call $\mathcal{A}$ irreducible [3].

Given a tensor $\mathcal{A}=(a_{i_{1}\cdots i_{m}})\in\mathbb {C}^{[m,n]}$, if there are $\lambda\in\mathbb{C}$ and $x=(x_{1},x_{2},\ldots,x_{n})^{T}\in\mathbb{C}\backslash\{0\}$ such that

$$\mathcal{A}x^{m-1}=\lambda x^{[m-1]}, $$

then *λ* is called an eigenvalue of $\mathcal{A}$ and *x* an eigenvector of $\mathcal{A}$ associated with *λ*, where $\mathcal{A}x^{m-1}$ is an *n* dimension vector whose *i*th component is

$$\bigl(\mathcal {A}x^{m-1}\bigr)_{i}=\sum _{i_{2},\ldots,i_{m}\in N} a_{ii_{2}\cdots i_{m}}x_{i_{2}}\cdots x_{i_{m}} $$

and

$$x^{[m-1]}=\bigl(x_{1}^{m-1},x_{2}^{m-1}, \ldots,x_{n}^{m-1}\bigr)^{T}. $$

If *λ* and *x* are all real, then *λ* is called an *H*-eigenvalue of $\mathcal {A}$ and *x* an *H*-eigenvector of $\mathcal{A}$ associated with *λ*; see [4, 5]. Moreover, the spectral radius $\rho(\mathcal{A})$ of $\mathcal{A}$ is defined as

$$\rho(\mathcal{A})=\max\bigl\{ \vert \lambda \vert :\lambda\in\sigma( \mathcal{A})\bigr\} , $$

where $\sigma(\mathcal{A})$ is the spectrum of $\mathcal{A}$, that is, $\sigma(\mathcal{A})=\{\lambda:\lambda \mbox{ is an eigenvalue of } \mathcal{A}\}$; see [3, 6].

A real tensor $\mathcal{A}$ is called an $\mathcal{M}$-tensor if there exist a nonnegative tensor $\mathcal{B}$ and a positive number $\alpha>\rho(\mathcal{B})$ such that $\mathcal {A}=\alpha\mathcal{I}-\mathcal{B}$, where $\mathcal{I}$ is called the unit tensor with its entries

$$ \delta_{i_{1}\cdots i_{m}}= \textstyle\begin{cases} 1& \mathrm{if}\ i_{1}=\cdots=i_{m}, \\ 0& \mathrm{otherwise}. \end{cases} $$

Denote by $\tau(\mathcal{A})$ the minimal value of the real part of all eigenvalues of an $\mathcal{M}$-tensor $\mathcal{A}$. Then $\tau (\mathcal{A})>0$ is an eigenvalue of $\mathcal{A}$ with a nonnegative eigenvector. If $\mathcal{A}$ is irreducible, then $\tau(\mathcal{A})$ is the unique eigenvalue with a positive eigenvector [7–9].

Recently, many people have focused on locating eigenvalues of tensors and using obtained eigenvalue inclusion theorems to determine the positive definiteness of an even-order real symmetric tensor or to give the lower and upper bounds for the spectral radius of nonnegative tensors and the minimum eigenvalue of $\mathcal {M}$-tensors. For details, see [1, 2, 10–14].

In 2015, Li *et al*. [1] proposed the following Brauer-type eigenvalue localization set for tensors.

### Theorem 1

[1], Theorem 6

*Let* $\mathcal{A}=(a_{i_{1}\cdots i_{m}})\in{\mathbb{C}}^{[m,n]}$. *Then*

$$\begin{aligned} \sigma(\mathcal{A})\subseteq\Delta(\mathcal{A})=\bigcup _{i,j\in {N},j\neq{i}}\Delta_{i}^{j}(\mathcal{A}), \end{aligned}$$

*where*

$$\begin{aligned} &\Delta_{i}^{j}(\mathcal{A})= \bigl\{ z\in\mathbb{C}:\bigl\vert (z-a_{i\cdots i}) (z-a_{j\cdots j})-a_{ij\cdots j}a_{ji\cdots i} \bigr\vert \leq \vert z-a_{j\cdots j}\vert r_{i}^{j}( \mathcal{A})+\vert a_{ij\cdots j}\vert r_{j}^{i}( \mathcal {A}) \bigr\} , \\ &r_{i}(\mathcal{A})=\sum_{\delta_{ii_{2}\cdots i_{m}}=0}\vert a_{ii_{2}\cdots i_{m}}\vert ,\qquad r_{i}^{j}(\mathcal{A})=\sum _{\substack{\delta_{ii_{2}\cdots i_{m}}=0,\\ \delta_{ji_{2}\cdots i_{m}}=0}}\vert a_{ii_{2}\cdots i_{m}}\vert =r_{i}(\mathcal{A})-\vert a_{ij\cdots{j}} \vert . \end{aligned}$$

To reduce computations, Huang *et al*. [2] presented an *S*-type eigenvalue localization set by breaking *N* into disjoint subsets *S* and *S̄*, where *S̄* is the complement of *S* in *N*.

### Theorem 2

[2], Theorem 3.1

*Let* $\mathcal{A}=(a_{i_{1}\cdots i_{m}})\in{\mathbb{C}}^{[m,n]}$, *S* *be a nonempty proper subset of* *N*, *S̄* *be the complement of* *S* *in* *N*. *Then*

$$\begin{aligned} \sigma(\mathcal{A})\subseteq\Delta^{S}(\mathcal{A}) = \biggl(\bigcup _{i\in S,j\in\bar{S}}\Delta_{i}^{j}(\mathcal{A}) \biggr)\cup \biggl(\bigcup_{i\in\bar{S},j\in S}\Delta_{i}^{j}( \mathcal{A}) \biggr). \end{aligned}$$

Based on Theorem 2, Huang *et al*. [2] obtained the following lower and upper bounds for the minimum eigenvalue of $\mathcal{M}$-tensors.

### Theorem 3

[2], Theorem 3.6

*Let* $\mathcal{A}=(a_{i_{1}\cdots i_{m}})\in\mathbb{R}^{[m, n]}$ *be an* $\mathcal{M}$-*tensor*, *S* *be a nonempty proper subset of* *N*, *S̄* *be the complement of* *S* *in* *N*. *Then*

$$\begin{aligned} \min \Bigl\{ \min_{i\in S}\max_{j\in\bar{S}}L_{ij}( \mathcal {A}), \min_{i\in\bar{S}}\max_{j\in S}L_{ij}( \mathcal {A}) \Bigr\} \leq\tau(\mathcal{A})\leq \max \Bigl\{ \max _{i\in S}\min_{j\in\bar{S}}L_{ij}(\mathcal {A}), \max_{i\in\bar{S}}\min_{j\in S}L_{ij}( \mathcal {A}) \Bigr\} , \end{aligned}$$

*where*

$$\begin{aligned} L_{ij}(\mathcal{A})=\frac{1}{2} \bigl\{ a_{i\cdots i}+a_{j\cdots j}-r_{i}^{j}( \mathcal{A})-\bigl[\bigl(a_{i\cdots i}-a_{j\cdots j}-r_{i}^{j}( \mathcal{A})\bigr)^{2}-4a_{ij\cdots j}r_{j}(\mathcal{A}) \bigr]^{\frac {1}{2}} \bigr\} . \end{aligned}$$

The main aim of this paper is to give a new eigenvalue inclusion set for tensors and prove that this set is tighter than those in Theorems 1 and 2 without considering the selection of *S*. And then we use this set to obtain new lower and upper bounds for the minimum eigenvalue of $\mathcal{M}$-tensors and prove that new bounds are sharper than those in Theorem 3.

## Main results

Now, we give a new eigenvalue inclusion set for tensors and establish the comparison between this set with those in Theorems 1 and 2.

### Theorem 4

*Let* $\mathcal{A}=(a_{i_{1}\cdots i_{m}})\in{\mathbb{C}}^{[m,n]}$. *Then*

$$\begin{aligned} \sigma(\mathcal{A})\subseteq\Delta^{\cap}(\mathcal{A})=\bigcup _{i\in N}\bigcap_{j\in N,j\neq i} \Delta_{i}^{j}(\mathcal{A}). \end{aligned}$$

### Proof

For any $\lambda\in\sigma(\mathcal{A})$, let $x=(x_{1},\ldots ,x_{n})^{T}\in{\mathbb{C}}^{n}\backslash\{0\}$ be an associated eigenvector, i.e.,

1 $$\begin{aligned} \mathcal{A}x^{m-1}=\lambda x^{[m-1]}. \end{aligned}$$

Let $\vert x_{p}\vert =\max\{\vert x_{i}\vert :i \in N\}$. Then $\vert x_{p}\vert >0$. For any $j\in N, j\neq p$, then from (1) we have

$$\begin{aligned} \lambda x_{p}^{m-1}=\mathop{\sum _{\delta_{pi_{2}\cdots i_{m}}=0,}}_{\delta _{ji_{2}\cdots i_{m}}=0}a_{pi_{2}\cdots i_{m}}x_{i_{2}}\cdots x_{i_{m}}+a_{p\cdots{p}}x_{p}^{m-1}+a_{pj\cdots{j}}x_{j}^{m-1} \end{aligned}$$

and

$$\begin{aligned} \lambda x_{j}^{m-1}=\mathop{\sum _{\delta_{ji_{2}\cdots i_{m}}=0,}}_{ \delta_{pi_{2}\cdots i_{m}}=0}a_{ji_{2}\cdots i_{m}}x_{i_{2}}\cdots x_{i_{m}}+a_{j\cdots{j}}x_{j}^{m-1}+a_{jp\cdots{p}}x_{p}^{m-1}, \end{aligned}$$

equivalently,

2 $$\begin{aligned} (\lambda-a_{p\cdots{p}})x_{p}^{m-1}-a_{pj\cdots{j}}x_{j}^{m-1}= \mathop{\sum_{\delta_{pi_{2}\cdots i_{m}}=0,}}_{\delta_{ji_{2}\cdots i_{m}}=0}a_{pi_{2}\cdots i_{m}}x_{i_{2}} \cdots x_{i_{m}} \end{aligned}$$

and

3 $$\begin{aligned} (\lambda-a_{j\cdots{j}})x_{j}^{m-1}-a_{jp\cdots{p}}x_{p}^{m-1}= \mathop{\sum_{\delta_{ji_{2}\cdots i_{m}}=0,}}_{\delta_{pi_{2}\cdots i_{m}}=0}a_{ji_{2}\cdots i_{m}}x_{i_{2}} \cdots x_{i_{m}}. \end{aligned}$$

Solving $x_{p}^{m-1}$ from (2) and (3), we get

$$\begin{aligned} &\bigl((\lambda-a_{p\cdots{p}}) (\lambda-a_{j\cdots{j}})-a_{pj\cdots {j}}a_{jp\cdots{p}} \bigr)x_{p}^{m-1}\\ &\quad = (\lambda-a_{j\cdots{j}})\mathop{\sum _{\delta_{pi_{2}\cdots i_{m}}=0,}}_{ \delta_{ji_{2}\cdots i_{m}}=0}a_{pi_{2}\cdots i_{m}}x_{i_{2}} \cdots x_{i_{m}} +a_{pj\cdots j}\mathop{\sum_{\delta_{ji_{2}\cdots i_{m}}=0,}}_{\delta _{pi_{2}\cdots i_{m}}=0}a_{ji_{2}\cdots i_{m}}x_{i_{2}} \cdots x_{i_{m}}. \end{aligned}$$

Taking absolute values and using the triangle inequality yields

$$\begin{aligned} &\bigl\vert (\lambda-a_{p\cdots{p}}) (\lambda-a_{j\cdots{j}})-a_{pj\cdots {j}}a_{jp\cdots{p}} \bigr\vert \vert x_{p}\vert ^{m-1}\\ &\quad\leq \vert \lambda-a_{j\cdots j}\vert r_{p}^{j}(\mathcal{A}) \vert x_{p}\vert ^{m-1}+\vert a_{pj\cdots j}\vert r_{j}^{p}(\mathcal{A})\vert x_{p}\vert ^{m-1}. \end{aligned}$$

Furthermore, by $\vert x_{p}\vert >0$, we have

$$\begin{aligned} \bigl\vert (\lambda-a_{p\cdots{p}}) (\lambda-a_{j\cdots{j}})-a_{pj\cdots {j}}a_{jp\cdots{p}} \bigr\vert \leq \vert \lambda-a_{j\cdots j}\vert r_{p}^{j}( \mathcal{A})+\vert a_{pj\cdots j}\vert r_{j}^{p}( \mathcal{A}), \end{aligned}$$

which implies that $\lambda\in\Delta_{p}^{j}(\mathcal{A})$. From the arbitrariness of *j*, we have $\lambda\in\bigcap_{j\in N, j\neq p}\Delta_{p}^{j}(\mathcal{A})$. Furthermore, we have $\lambda\in\bigcup_{i\in N}\bigcap_{j\in N, j\neq i}\Delta _{i}^{j}(\mathcal{A})$. The conclusion follows. □

Next, a comparison theorem is given for Theorems 1, 2 and 4.

### Theorem 5

*Let* $\mathcal{A}=(a_{i_{1}\cdots i_{m}})\in{\mathbb{C}}^{[m,n]}$, *S* *be a nonempty proper subset of* *N*. *Then*

$$\begin{aligned} \Delta^{\cap}(\mathcal{A})\subseteq\Delta^{S}(\mathcal{A}) \subseteq\Delta (\mathcal{A}). \end{aligned}$$

### Proof

By Theorem 3.2 in [2], $\Delta^{S}(\mathcal{A})\subseteq\Delta(\mathcal{A})$. Here, only $\Delta^{\cap}(\mathcal{A})\subseteq\Delta^{S}(\mathcal{A})$ is proved. Let $z\in\Delta^{\cap}(\mathcal{A})$, then there exists some $i_{0}\in N$ such that $z\in\Delta_{i_{0}}^{j}(\mathcal {A}),\forall j\in N, j\neq i_{0}$. Let *S̄* be the complement of *S* in *N*. If $i_{0}\in S$, then taking $j\in\bar{S}$, obviously, $z\in\bigcup_{i_{0}\in S,j\in\bar{S}}\Delta_{i_{0}}^{j}(\mathcal{A})\subseteq \Delta^{S}(\mathcal{A})$. If $i_{0}\in\bar{S}$, then taking $j\in S$, obviously, $z\in\bigcup_{i_{0}\in\bar{S},j\in S}\Delta_{i_{0}}^{j}(\mathcal {A})\subseteq\Delta^{S}(\mathcal{A})$. The conclusion follows. □

### Remark 1

Theorem 5 shows that the set $\Delta^{\cap}(\mathcal{A})$ in Theorem 4 is tighter than those in Theorems 1 and 2, that is, $\Delta^{\cap}(\mathcal{A})$ can capture all eigenvalues of $\mathcal {A}$ more precisely than $\Delta(\mathcal{A})$ and $\Delta^{S}(\mathcal{A})$.

In the following, we give new lower and upper bounds for the minimum eigenvalue of $\mathcal{M}$-tensors.

### Theorem 6

*Let* $\mathcal{A}=(a_{i_{1}\cdots i_{m}})\in\mathbb{R}^{[m, n]}$ *be an irreducible* $\mathcal{M}$-*tensor*. *Then*

$$\begin{aligned} \min_{i\in N}\max_{j\neq i}L_{ij}( \mathcal{A})\leq\tau (\mathcal{A})\leq \max_{i\in N}\min _{j\neq i}L_{ij}(\mathcal{A}). \end{aligned}$$

### Proof

Let $x=(x_{1},x_{2},\ldots,x_{n})^{T}$ be an associated positive eigenvector of $\mathcal{A}$ corresponding to $\tau(\mathcal{A})$, i.e.,

4 $$\begin{aligned} \mathcal{A}x^{m-1}=\tau(\mathcal{A}) x^{[m-1]}. \end{aligned}$$

(I) Let $x_{q}=\min\{x_{i}:i\in N\}$. For any $j\in N, j\neq q$, we have by (4) that

$$\tau(\mathcal{A})x_{q}^{m-1}=\mathop{\sum _{\delta_{qi_{2} \cdots i_{m}}=0,}}_{\delta_{ji_{2} \cdots i_{m}}=0}a_{qi_{2}\cdots i_{m}}x_{i_{2}}\cdots x_{i_{m}}+a_{q\cdots q}x_{q}^{m-1}+a_{qj\cdots j}x_{j}^{m-1} $$

and

$$\tau(\mathcal{A})x_{j}^{m-1}=\mathop{\sum _{\delta_{ji_{2} \cdots i_{m}}=0,}}_{\delta_{qi_{2} \cdots i_{m}}=0}a_{ji_{2}\cdots i_{m}}x_{i_{2}}\cdots x_{i_{m}}+a_{j\cdots j}x_{j}^{m-1}+a_{jq\cdots q}x_{q}^{m-1}, $$

equivalently,

5 $$\begin{aligned} \bigl(\tau(\mathcal{A})-a_{q\cdots q}\bigr)x_{q}^{m-1}-a_{qj\cdots j}x_{j}^{m-1}= \mathop{\sum_{\delta_{qi_{2} \cdots i_{m}}=0,}}_{\delta_{ji_{2} \cdots i_{m}}=0}a_{qi_{2}\cdots i_{m}}x_{i_{2}} \cdots x_{i_{m}} \end{aligned}$$

and

6 $$\begin{aligned} \bigl(\tau(\mathcal{A})-a_{j\cdots j}\bigr)x_{j}^{m-1}-a_{jq\cdots q}x_{q}^{m-1}= \mathop{\sum_{\delta_{ji_{2} \cdots i_{m}}=0,}}_{\delta_{qi_{2} \cdots i_{m}}=0}a_{ji_{2}\cdots i_{m}}x_{i_{2}} \cdots x_{i_{m}}. \end{aligned}$$

Solving $x_{q}^{m-1}$ by (5) and (6), we get

$$\begin{aligned} &\bigl(\bigl(\tau(\mathcal{A})-a_{q\cdots q}\bigr) \bigl(\tau( \mathcal{A})-a_{j\cdots j}\bigr)-a_{qj\cdots j}a_{jq\cdots q} \bigr)x_{q}^{m-1}\\ &\quad=\bigl(\tau(\mathcal{A})-a_{j\cdots j} \bigr)\mathop{\sum_{\delta_{qi_{2} \cdots i_{m}}=0,}}_{\delta_{ji_{2} \cdots i_{m}}=0}a_{qi_{2}\cdots i_{m}}x_{i_{2}} \cdots x_{i_{m}} +a_{qj\cdots j}\mathop{\sum_{\delta_{ji_{2} \cdots i_{m}}=0,}}_{\delta_{qi_{2} \cdots i_{m}}=0}a_{ji_{2}\cdots i_{m}}x_{i_{2}} \cdots x_{i_{m}}. \end{aligned}$$

From Theorem 2.1 in [9], we have $\tau(\mathcal{A})\leq\min_{i\in N}a_{i\cdots i}$ and

$$\begin{aligned} &\bigl(\bigl(a_{q\cdots q}-\tau(\mathcal{A})\bigr) \bigl(a_{j\cdots j}- \tau(\mathcal{A})\bigr)-a_{qj\cdots j}a_{jq\cdots q}\bigr)x_{q}^{m-1} \\ &\quad=\bigl(a_{j\cdots j}-\tau(\mathcal{A})\bigr)\mathop{\sum _{\delta_{qi_{2} \cdots i_{m}}=0,}}_{\delta_{ji_{2} \cdots i_{m}}=0}\vert a_{qi_{2}\cdots i_{m}}\vert x_{i_{2}}\cdots x_{i_{m}} +\vert a_{qj\cdots j}\vert \mathop{ \sum_{\delta_{ji_{2} \cdots i_{m}}=0,}}_{\delta _{qi_{2} \cdots i_{m}}=0} \vert a_{ji_{2}\cdots i_{m}}\vert x_{i_{2}}\cdots x_{i_{m}}. \end{aligned}$$

Hence,

$$\begin{aligned} &\bigl(\bigl(a_{q\cdots q}-\tau(\mathcal{A})\bigr) \bigl(a_{j\cdots j}- \tau(\mathcal{A})\bigr)-\vert a_{qj\cdots j}\vert \vert a_{jq\cdots q} \vert \bigr)x_{q}^{m-1} \\ &\quad \geq\bigl(a_{j\cdots j}-\tau( \mathcal{A})\bigr)\mathop{\sum_{\delta_{qi_{2} \cdots i_{m}}=0,}}_{\delta_{ji_{2} \cdots i_{m}}=0} \vert a_{qi_{2}\cdots i_{m}}\vert x_{q}^{m-1} +\vert a_{qj\cdots j}\vert \mathop{\sum_{\delta_{ji_{2} \cdots i_{m}}=0,}}_{\delta_{qi_{2} \cdots i_{m}}=0} \vert a_{ji_{2}\cdots i_{m}}\vert x_{q}^{m-1}. \end{aligned}$$

From $x_{q}>0$, we have

$$\begin{aligned} &\bigl(a_{q\cdots q}-\tau(\mathcal{A})\bigr) \bigl(a_{j\cdots j}-\tau( \mathcal{A})\bigr)-\vert a_{qj\cdots j}\vert \vert a_{jq\cdots q}\vert \\ &\quad \geq\bigl(a_{j\cdots j}-\tau(\mathcal{A})\bigr)\mathop{\sum _{\delta_{qi_{2} \cdots i_{m}}=0,}}_{\delta_{ji_{2} \cdots i_{m}}=0}\vert a_{qi_{2}\cdots i_{m}}\vert +\vert a_{qj\cdots j}\vert \mathop{\sum_{\delta_{ji_{2} \cdots i_{m}}=0,}}_{\delta_{qi_{2} \cdots i_{m}}=0} \vert a_{ji_{2}\cdots i_{m}}\vert \\ &\quad=\bigl(a_{j\cdots j}-\tau(\mathcal{A})\bigr)r_{q}^{j}( \mathcal{A})+\vert a_{qj\cdots j}\vert r_{j}^{q}( \mathcal{A}), \end{aligned}$$

equivalently,

$$\bigl(a_{q\cdots q}-\tau(\mathcal{A})\bigr) \bigl(a_{j\cdots j}-\tau( \mathcal{A})\bigr)-\bigl(a_{j\cdots j}-\tau(\mathcal{A})\bigr)r_{q}^{j}( \mathcal{A})-\vert a_{qj\cdots j}\vert r_{j}(\mathcal{A})\geq0, $$

that is,

$$\tau(\mathcal{A})^{2}-\bigl(a_{q\cdots q}+a_{j\cdots j}-r_{q}^{j}( \mathcal{A})\bigr)\tau(\mathcal{A})+a_{q\cdots q}a_{j\cdots j}-a_{j\cdots j}r_{q}^{j}( \mathcal{A})+a_{qj\cdots j}r_{j}(\mathcal{A})\geq0. $$

Solving for $\tau(\mathcal{A})$ gives

$$\tau(\mathcal{A})\leq\frac{1}{2} \bigl\{ a_{q\cdots q}+a_{j\cdots j}-r_{q}^{j}( \mathcal{A})- \bigl[\bigl(a_{q\cdots q}-a_{j\cdots j}-r_{q}^{j}( \mathcal{A})\bigr)^{2}-4a_{qj\cdots j}r_{j}(\mathcal{A}) \bigr]^{\frac {1}{2}} \bigr\} =L_{qj}(\mathcal{A}). $$

For the arbitrariness of *j*, we have $\tau(\mathcal{A})\leq\min_{j\neq q}L_{qj}(\mathcal{A})$. Furthermore, we have

$$\begin{aligned} \tau(\mathcal{A})\leq\max_{i\in N}\min_{j\neq i}L_{ij}( \mathcal{A}). \end{aligned}$$

(II) Let $x_{p}=\max\{x_{i}:i\in N\}$. Similar to (I), we have

$$\begin{aligned} \tau(\mathcal{A})\geq\min_{i\in N}\max_{j\neq i}L_{ij}( \mathcal{A}). \end{aligned}$$

The conclusion follows from (I) and (II). □

Similar to the proof of Theorem 3.6 in [2], we can extend the results of Theorem 6 to a more general case.

### Theorem 7

*Let* $\mathcal{A}=(a_{i_{1}\cdots i_{m}})\in\mathbb{R}^{[m, n]}$ *be an* $\mathcal{M}$-*tensor*. *Then*

$$\begin{aligned} \min_{i\in N}\max_{j\neq i}L_{ij}( \mathcal{A})\leq\tau (\mathcal{A})\leq \max_{i\in N}\min _{j\neq i}L_{ij}(\mathcal{A}). \end{aligned}$$

By Theorems 3, 6 and 7 in [13], the following comparison theorem is obtained easily.

### Theorem 8

*Let* $\mathcal{A}=(a_{i_{1}\cdots i_{m}})\in\mathbb{R}^{[m, n]}$ *be an* $\mathcal{M}$-*tensor*, *S* *be a nonempty proper subset of* *N*, *S̄* *be the complement of* *S* *in* *N*. *Then*

$$\begin{aligned} \min_{i\in N}R_{i}(\mathcal{A})&\leq \min _{j\neq i}L_{ij}(\mathcal{A})\leq \min \Bigl\{ \min _{i\in S}\max_{j\in\bar{S}}L_{ij}(\mathcal {A}), \min_{i\in\bar{S}}\max_{j\in S}L_{ij}( \mathcal {A}) \Bigr\} \leq \min_{i\in N}\max_{j\neq i}L_{ij}( \mathcal{A}) \\ &\leq \max_{i\in N}\min_{j\neq i}L_{ij}( \mathcal{A}) \leq \max \Bigl\{ \max_{i\in S}\min _{j\in\bar{S}}L_{ij}(\mathcal {A}), \max_{i\in\bar{S}} \min_{j\in S}L_{ij}(\mathcal {A}) \Bigr\} , \end{aligned}$$

*where* $R_{i}(\mathcal{A})=\sum_{i_{2},\ldots,i_{m}\in N}a_{ii_{2}\cdots i_{m}}$.

### Remark 2

Theorem 8 shows that the bounds in Theorem 7 are shaper than those in Theorem 3, Theorem 2.1 of [9] and Theorem 4 of [13] without considering the selection of *S*, which is also the advantage of our results.

## Numerical examples

In this section, two numerical examples are given to verify the theoretical results.

### Example 1

Let $\mathcal{A}=(a_{ijk})\in\mathbb{R}^{[3, 4]}$ be an irreducible $\mathcal{M}$-tensor with elements defined as follows:

$$\begin{aligned} &\mathcal{A}(:,:,1)= \begin{pmatrix} 62&-3&-4&-2\\ -4&-2&-2&-1\\ -3&-1&-3&-3\\ -3&-3&-2&-2 \end{pmatrix} ,\qquad \mathcal{A}(:,:,2)= \begin{pmatrix} 0&-4&-3&-3\\ -1&28&-2&-2\\ -1&-2&-2&-4\\ -2&-2&-3&-1 \end{pmatrix}, \\ &\mathcal{A}(:,:,3)= \begin{pmatrix} -2&-1&-2&-1\\ -1&-1&-1&-2\\ -2&-4&63&-4\\ -4&-4&-2&-2 \end{pmatrix},\qquad \mathcal{A}(:,:,4)= \begin{pmatrix} -4&-2&-2&-1\\ -1&-2&-3&-1\\ -2&-3&-3&-2\\ -2&-2&-4&61 \end{pmatrix}. \end{aligned}$$

By Theorem 2.1 in [9], we have

$$2=\min_{i\in N}R_{i}(\mathcal{A})\leq\tau(\mathcal{A}) \leq\min\Bigl\{ \max_{i\in N}R_{i}(\mathcal{A}),\min _{i\in N}a_{i\cdots i}\Bigr\} =28. $$

By Theorem 4 in [13], we have

$$\tau(\mathcal{A})\geq\min_{j\neq i}L_{ij}( \mathcal{A})=2.3521. $$

By Theorem 3, we have

$$\begin{aligned} &\mbox{if }S=\{1\},\bar{S}=\{2,3,4\},\quad 3.6685\leq\tau(\mathcal{A})\leq 24.2948;\\ &\mbox{if }S=\{2\},\bar{S}=\{1,3,4\},\quad 3.6685\leq\tau(\mathcal{A})\leq 19.7199;\\ &\mbox{if }S=\{3\},\bar{S}=\{1,2,4\},\quad 2.3569\leq\tau(\mathcal{A})\leq 27.7850;\\ &\mbox{if }S=\{4\},\bar{S}=\{1,2,3\},\quad 2.3521\leq\tau(\mathcal{A})\leq 27.8536;\\ &\mbox{if }S=\{1,2\},\bar{S}=\{3,4\},\quad 2.3569\leq\tau(\mathcal{A})\leq 27.7850;\\ &\mbox{if }S=\{1,3\},\bar{S}=\{2,4\},\quad 3.6685\leq\tau(\mathcal{A})\leq 23.0477;\\ &\mbox{if }S=\{1,4\},\bar{S}=\{2,3\},\quad 3.6685\leq\tau(\mathcal{A})\leq 23.9488. \end{aligned}$$

By Theorem 7, we have

$$3.6685\leq\tau(\mathcal{A})\leq19.7199. $$

In fact, $\tau(\mathcal{A})=14.4049$. Hence, this example verifies Theorem 8 and Remark 2, that is, the bounds in Theorem 7 are sharper than those in Theorem 3, Theorem 2.1 of [9] and Theorem 4 of [13] without considering the selection of *S*.

### Example 2

Let $\mathcal{A}=(a_{ijkl})\in\mathbb{R}^{[4, 2]}$ be an $\mathcal {M}$-tensor with elements defined as follows:

$$a_{1111}=6,\qquad a_{1222}=-1,\qquad a_{2111}=-2,\qquad a_{2222}=5, $$

other $a_{ijkl}=0$. By Theorem 7, we have

$$4\leq\tau(\mathcal{A})\leq4. $$

In fact, $\tau(\mathcal{A})=4$.

## Conclusions

In this paper, we give a new eigenvalue inclusion set for tensors and prove that this set is tighter than those in [1, 2]. As an application, we obtain new lower and upper bounds for the minimum eigenvalue of $\mathcal{M}$-tensors and prove that the new bounds are sharper than those in [2, 9, 13]. Compared with the results in [2], the advantage of our results is that, without considering the selection of *S*, we can obtain a tighter eigenvalue localization set for tensors and sharper bounds for the minimum eigenvalue of $\mathcal{M}$-tensors.
